# Permanent Union of the Maxillary Bones

**Published:** 1869-03

**Authors:** 


### ARTICLE III.

### *Permanent Union of the Maxillary Bones.*

Prof. Edward Warren of the *Washington University of Medicine*, Baltimore, relates the following case of Miss D. aged eighteen, a native of Virginia, who was recently brought to Baltimore by Dr. H. M. Grant, Demonstrator of Operative Dentistry in the *Baltimore College of Dental Surgery*, to secure the professional services of Prof. Warren.

" A very singular condition of things presented itself, from which much deformity and great inconvenience resulted. As the effect of ptyalism which occurred at the age of *four* years, the alveolar process of the superior maxillary had been destroyed, and an osseous band developed, which encased the lower teeth, and attached itself to the alveolar process of the inferior maxillary, binding the two bones firmly and permanently together.

The tissues of the cheek were greatly atrophied, while the integument was adherent to the bony structures beneath it being held down by bands of organized lymph in this position.

The *inferior* maxillary could not be separated from the *superior* to the smallest extent; and for thirteen years the patient had been nourished only by liquids and such articles of a semi-solid consistence as could be sucked or pushed into the cavity of the mouth through openings between the teeth on the opposite side.

As a natural effect of this mode of feeding, she was delicate and anæmic, though no disease of any important organ existed. Upon examination Prof. Warren ascertained that there was no anchylosis of the temporo-maxillary joint, and that the pterygoid muscles were neither paralysed nor degenerated. Relying upon these facts, together with the integrity of the masseter and temporal muscles of the healthy side, he concluded that it was possible to restore the movements of the inferior maxillary and to relieve the patient from her condition of embarrassment, by an operation, which was performed in the following manner: Seating the patient in a dentist's chair, and placing her in a semi-recumbent posture, chloroform was administered. A probe pointed bistoury was then introduced through the mouth between the cheek and the bony structures, and the adhering tissues carefully separated from their attachments, taking special care not to divide the facial artery which was much exposed. The upper bicuspid tooth on the affected side was then extracted, and an attack made upon the bony deposit by which the maxillary bones were bound together. This osseous band was found to be excessively hard, but by employing first a saw and then a strong pair of forceps, the first of the lower teeth embeded in it, was reached, and the tooth extracted. This gave still more space to work in, and enabled the Professor, after much labor, to reach the second one of the encased teeth and to remove it. These different steps were repeated until there was room enough for the introduction of Liston's Bone Forceps, when the whole of the intervening mass was cut through, and the inferior maxillary entirely released. A spatula was then passed into the mouth, and the jaw separated widely by the employment of considerable force. The chloroform was then discontinued, and, so soon as the anæsthetic condition had passed off, it was ascertained that the patient could open and close her jaws at will. Lint was then introduced between the divided bony surfaces, and also in the cavity of the jaw, between the tissues and the osseous structures from which they had been detached, and the patient removed to her bed. Upon examination, it was ascertained that she had been kept under the influence of chloroform for two hours and twenty-five minutes, so tardily was the osseous band divided, in consequence of its extreme density, and of the disadvantages under which the operator labored in assailing it. Not an unfavorable symptom presented itself from this time forward. By the frequent introduction of lint in the manner stated above, the divided surfaces were kept from uniting again ; and in a short time the patient returned to her home being able to open her mouth and to masticate food without difficulty. The improvement in her power of articulating words, as well as in general health, was most marked and decided, while the deformity was almost entirely removed."

